# The polyAT, intronic IVS11-6 and Lys939Gln *XPC* polymorphisms are not associated with transitional cell carcinoma of the bladder

**DOI:** 10.1038/sj.bjc.6602616

**Published:** 2005-05-10

**Authors:** S C Sak, J H Barrett, A B Paul, D T Bishop, A E Kiltie

**Affiliations:** 1 Molecular Radiobiology Group, Cancer Research UK Clinical Centre in Leeds, St James's University Hospital, Leeds LS9 7TF, UK; 2 Genetic Epidemiology Division, Cancer Research UK Clinical Centre in Leeds, St James's University Hospital, Leeds LS9 7TF, UK; 3 Department of Urology, St James's University Hospital, Leeds LS9 7TF, UK

**Keywords:** bladder cancer, transitional cell carcinoma, Xeroderma Pigmentosum Group C (XPC), polymorphisms

## Abstract

Chemical carcinogens from cigarette smoking and occupational exposure are risk factors for bladder transitional cell carcinoma (TCC). The Xeroderma Pigmentosum Group C (*XPC*) gene is essential for repair of bulky adducts from carcinogens. The Xeroderma Pigmentosum Group C gene polymorphisms may alter DNA repair capacity (DRC), thus giving rise to genetic predisposition to bladder cancer. Recent studies have demonstrated linkage disequilibrium between three polymorphisms in the *XPC* gene (polyAT, IVS11-6 and Lys939Gln) and these have been shown to influence the DRC, as well as to be associated with bladder cancer risk. We analysed all three *XPC* polymorphisms in 547 bladder TCC patients and 579 cancer-free controls to investigate the association between these polymorphisms and bladder cancer susceptibility, and we also attempted to assess gene–environmental interactions. We confirmed strong linkage disequilibrium among the polymorphisms (Lewontin's *D*′>0.99). Using logistic regression adjusting for smoking, occupational and family history, neither the heterozygote nor the homozygote variants of these polymorphisms were associated with increased bladder cancer risk (adjusted odds ratio [95% confidence interval] for heterozygote 0.82 [0.63–1.07], 0.82 [0.63–1.08] and 0.83 [0.63–1.08] for PolyAT, IVS11-6 and Lys939Gln, respectively and homozygote variant, 0.98 [0.68–1.42], 0.99 [0.69–1.43] and 1.01 [0.70–1.46]). Moreover, we did not find any significant interaction between these *XPC* polymorphisms and environmental exposure to cigarette smoking and occupational carcinogens.

Transitional cell carcinoma (TCC) of the bladder is the fourth commonest cancer in men and eighth in women in the USA and UK (Greenlee *et al*, 2000; Office of National Statistics, 2000). The two established risk factors for TCC are chemical carcinogens from cigarette smoking and occupation exposure (Zeegers *et al*, 2000; Kogevinas *et al*, 2003). Bulky DNA adducts formed by these carcinogens are repaired by nucleotide excision repair (NER). Failure to repair this DNA damage can cause mutations in oncogenes and tumour suppressor genes resulting in tumour formation. The xeroderma pigmentosum group C (*XPC*) protein plays an important role in the initiation of this pathway. Polymorphisms in the *XPC* gene may alter NER capacity, thus giving rise to genetic predisposition to bladder cancer.

Recently, Khan *et al* (2000) discovered a common *XPC* polyAT (XPC-PAT) polymorphism in intron 9, which involves deletion of five base pairs (bp) and insertion of an 83 bp AT repeat. The XPC-PAT was found to be in strong linkage disequilibrium (LD) with the *XPC* coding single-nucleotide polymorphism (SNP) Lys939Gln in exon 15 and also an intronic SNP (IVS11-6) in intron 11 that effects alternative splicing and hence alters protein function (Khan *et al*, 2002). Although the abnormal splice variant resulted in reduced DNA repair capacity (DRC) *in vitro* using an allele-specific post-UV plasmid host cell reactivation assay (Khan *et al*, 2002), the variant XPC 939Gln allele was not associated with reduced DRC (Khan *et al*, 2000). However, in a recent case–control study, homozygotes for the 939Gln allele were found to have a significantly increased risk of bladder cancer (Sanyal *et al*, 2004). In addition, the homozygous variant XPC-PAT insertion (‘+/+’) has been associated with an increased risk of squamous cell carcinoma of the head and neck (Shen *et al*, 2001), and in lymphocytes from normal subjects homozygotes for XPC-PAT had reduced DRC (Qiao *et al*, 2002).

We therefore hypothesised that the *XPC* polymorphisms XPC-PAT, IVS11-6 and Lys939Gln have a role in the aetiology of TCC of the bladder through modification of risk produced by smoking and occupational exposure. We conducted a case–control study to examine the LD between all three polymorphisms and their association with bladder TCC risk. We also investigated gene–environment interactions between the *XPC* polymorphisms and smoking and occupational exposure to chemical carcinogens.

## MATERIALS AND METHODS

### Study population

Local ethical approval was obtained from the Leeds Teaching Hospitals Local Research Ethics Committee (LREC) for this study. Written informed consent was obtained from all of the participating subjects.

In total, 626 patients were admitted to our institution for bladder tumour surgery between August 2002 to April 2004. Eligibility criteria for cases were patients with pathologically confirmed TCC in the tumour specimen obtained from biopsy or surgical resection, age more than 18 years and mental competence to give informed consent. Exclusion criteria were patients with non-TCC tumours (*n*=46), mental incompetence such as dementia (*n*=6), patients who had had a blood transfusion less than 1 month before surgery (*n*=23). In all, 92% of eligible patients were recruited (547 patients out of 549 eligible subjects); only two patients declined to participate in this study. Controls were subjects who were cancer free and had no symptom of haematuria (blood in urine). They were frequency matched by age and sex but no attempt was made at one-to-one matching of cases and controls. The controls (*n*=579) were recruited from two sources: community-based (*n*=227) from a previous molecular epidemiology study (Barrett *et al*, 2003), in the same region and hospital-based (*n*=352) from individuals who attended the ophthalmology and otolaryngology clinics in the same institution. Overall, the refusal rate of participation from the controls was approximately 20%. After informed consent was obtained, a blood specimen was collected from each subject.

### Data collection

Each subject underwent a 15–20 min interview with investigators and completed a structured health questionnaire regarding smoking, occupation and family history. Smoking status was recorded as nonsmoker, exsmoker or current smoker, and smoking dose was measured in pack years. (One pack-year was defined as smoking 20 cigarettes per day for 1 year or any pattern leading to the same total exposure). Occupational exposure was defined as participating in occupations involving the rubber industry, the plastics industry, laboratories, printing, paints, dyes or diesel fumes. Positive family history was defined as reporting having any first- or second-degree relative diagnosed with bladder cancer. Histopathological information regarding tumour stage and grade was determined according to the TNM (tumour-nodes-metastasis) staging system and data were obtained from patient medical notes and the hospital pathology report.

### Genotyping

Genomic DNA was extracted from blood samples collected from patients with TCC and control subjects using the modified salt precipitation method Puregene kit (Nottingham, UK) in the Regional Genetics Laboratory, Leeds. The method of Khan *et al* (2000) was used to amplify the XPC polyAT polymorphism. We fluorescently labelled the forward primer used by Khan *et al* to allow gel-free genotyping using a Genetic Analyser 3100 (Applied Biosystems, Foster City, CA, USA). Genotyping for SNP Lys939Gln and IVS11-6 were performed in the Cancer Research UK Genotyping Facility, Oxford, using the allelic discrimination 5′ nuclease assay (Taqman) without prior knowledge of the subject's clinical status. Primer and probe sets were designed and manufactured using Applied Biosystems ‘Assay-by-Design’ custom service (Applera, Austria). The primers and probe sets used for Lys939Gln were: forwards primer (5′AGCAGCTTCCCACCTGTTC 3′), reverse primer (5′ GTGGGTGCCCCTCTAGTG 3′), VIC probe (5′ CTCACAGCTTCTCAAAT 3′) and FAM probe (5′ CACAGCTGCTCAAAT 3′), and for IVS11-6 were forward primer (5′ GAGGTACACATTCCCAAACTCGTT 3′), reverse primer (5′ GCTGGCCAAATGCTGACTTG 3′), VIC probe (5′ CACCCGCCACAGGT 3′) and FAM probe (5′ TCACCCGACACAGGT 3′). Each PCR reaction was carried out in a 384-well MicroAmp optical plate (ABI) using a total volume of 10 *μ*l containing 5 *μ*l of 2 × Taqman universal master mix, 20 *μ*M of sequence detection primers, 5 *μ*M of allele-specific hybridisation probes and 10 ng of genomic DNA. Thermal cycling parameters were: 2 min at 50° and 10 min at 95° for primary denaturation, followed by 40 cycles of 15 s at 95° and 1 min at 60°. Once the thermal cycling had been completed, the plate was transferred to an Applied Biosystems 7900HT sequence detection system and the fluorescence in each reaction was read. Using the graphical view within the SDS software (ABI), each group of alleles could be manually selected and each sample designated as homozygous wild-type, heterozygous or homozygous variant. As a quality control, 5% of the samples (*n*=57) were reanalysed, and results were identical for all samples (100% concordance).

### Statistical analysis

All data were analysed using STATA version 8 (StataCorp LP, Texas). Hardy–Weinberg equilibrium analyses were performed to compare observed and expected genotype frequencies using a chi-squared goodness-of-fit test. The LD between polymorphisms was estimated by pairwise Lewontin's *D*′ using the STATA *pwld* function (〈http://www-gene.cimr.cam.ac.uk/clayton/〉). The odds ratio (OR) and 95% confidence interval (CI) for bladder cancer associated with each genotype were calculated using logistic regression analysis and were adjusted for established bladder cancer risk factors (age, sex, cigarette smoking, occupational exposure and family history).

## RESULTS

### Subject characteristics

The demographic characteristics of the 547 cases and 579 controls are shown in Table 1. The majority of the subjects (98.6%) were Caucasian and 83.5% were born in the Yorkshire region, UK. Despite attempts to match on sex, there were slightly more men in the case group (71%) than the control group (65%). As expected, the smoking prevalence among cases was higher than in controls. Similarly, cases were more likely to have had occupational exposure and a positive family history of bladder cancer compared to controls (*P*<0.001 and 0.02, respectively).

### XPC polymorphisms and the risk of bladder TCC

Overall, genotyping was successful in more than 98% of samples (99.6, 97.9 and 97.1% for XPC-PAT, IVS11-6 and Lys939Gln, respectively). The control genotype distribution for all three polymorphisms was in Hardy–Weinberg equilibrium (*P*=0.76, 0.63 and 0.67 for XPC-PAT, IVS11-6 and Lys939Gln, respectively). We also compared the genotype distributions between the community and the hospital controls and found no significant difference in genotype distribution between the two control groups (*P*=0.18, 0.13, 0.12 for XPC-PAT, IVS11-6 and Lys939Gln, respectively). Therefore, we combined the controls to increase our power to detect an association between the polymorphisms and bladder TCC risk. Among 1086 subjects with complete genotypes for all three polymorphisms, the genotype distribution and LD are shown in Table 2. The pairwise Lewontin's *D*′ among the three polymorphisms were all at least 0.99.

The frequencies of the XPC-PAT, IVS11-6 and Lys939Gln in bladder TCC cases and the controls are shown in Table 3. To evaluate the risk of bladder TCC according to genotype, logistic regression analysis was conducted with an adjustment for age, gender, smoking, occupation and family history of bladder cancer (Table 3). There was no association between any of the three polymorphisms and bladder TCC risk. We also analysed the association of these polymorphisms in white subjects only, and the result was very similar. None of the three polymorphisms were significantly associated with increased bladder TCC risk (chi square, *P*=0.14, 0.17, 0.17 for XPC-PAT, IVS11-6 and Lys939Gln, respectively). We also found no evidence of interactions between the genotypes of any of the three polymorphisms and smoking exposure or occupational exposure (*P*-value for departure from multiplicative joint effect=0.75 and 0.82 for smoking and occupational exposure). We present data for XPC-PAT only as results for the other two polymorphisms were very similar (Table 4 and data not shown).

## DISCUSSION

To our knowledge, this is the largest case–control study of all three polymorphisms of the *XPC* gene in bladder cancer, and it involved an ethnically homogeneous population. With a sample size of 547 cases and 579 controls, we had an 83% power (two-sided test, *P*=0.05) to detect an OR of 1.6 for the homozygote variant compared with other genotypes. Our study was limited by potential selection bias since we included both community and hospital controls. However, the similarity of the genotype distributions between the two control groups makes it unlikely that the use of controls initially recruited for another study has influenced our conclusions. We also cannot exclude any recall bias for smoking and occupational exposure.

From our data, we observed no association between the three XPC polymorphisms and bladder TCC risk. Sanyal *et al*, in a study of 304 cases and 246 controls, estimated an OR of 1.97 (95% CI 1.10–3.57) for 939 Gln/Gln homozygotes compared with those with other genotypes. Although our study was larger and had sufficient power to replicate this, we found no evidence to support the finding of increased bladder cancer risk. This discrepancy could be due to differences in the populations studied. Alternatively, the earlier finding could be a false positive result. Wacholder *et al* (2004) have shown that, unless the prior probability of an association is high, a large proportion of genetic associations showing marginal statistical significance in studies of low to moderate power will be false positive results. Of interest, a recent follow-up study of 288 superficial bladder cancer patients showed no association between XPC-PAT or Lys939Gln variants and increased risk of tumour recurrence (Gu *et al*, 2005).

We observed no interactions between the three XPC polymorphisms and smoking or occupational exposure by stratifying by smoking status (nonsmokers *vs* ex or current smokers) and by occupational exposure (yes or no). However, our study is unable to rule out more complex interactions with environmental exposures.

In conclusion, we found the XPC polymorphisms XPC-PAT, Lys939Gln and IVS11-6 to be in LD (*D*′⩾0.99), and none of the polymorphisms was associated with increased risk of bladder TCC.

## Figures and Tables

**Table 1:** Characteristics of study subjects

**Risk factors**	**Cases (%)**	**Controls (%)**	*P*-value*
*Age at interview (years)*			
⩽60	54 (13.0)	75 (9.9)	
61–70	142 (24.3)	141 (26.0)	
71–80	212 (39.0)	226 (38.7)	
⩾80	139 (23.7)	137 (25.4)	0.4
			
*Gender*			
Female	159 (29.1)	200 (34.5)	
Male	388 (70.9)	379 (65.5)	0.05
			
*Race*			
White	539 (98.5)	571(98.6)	
Other	8 (1.5)	8 (1.4)	0.91
			
*Smoking status* a			
Never	121 (22.2)	195 (33.7)	
Ex	293 (53.7)	288 (49.7)	
Current	132 (24.1)	96 (16.6)	<0.001
			
*Number of years smoked* a			
0	121 (22.2)	195 (33.7)	
1–20	81 (14.8)	108 (18.6)	
21–40	136 (24.9)	132 (22.8)	
>40	208 (38.1)	144 (24.9)	<0.001
			
Number of pack-years^a^			
0	121 (22.2)	195 (33.7)	
⩽25.5	195 (35.7)	202 (34.9)	
>25.5	216 (39.6)	176 (30.4)	
Pipe smoker	14 (2.5)	6 (1.0)	<0.001
			
*Occupational exposure*			
No	397 (72.6)	482 (83.2)	
Yes	150 (27.4)	97 (16.8)	<0.001
			
*Family history of bladder cancer*			
No	521 (95.2)	566 (97.8)	
Yes	26 (4.8)	13 (2.2)	0.02

* All *P*-values were calculated by chi-squared testing of contingency tables.

a One case had inadequate smoking information.

**Table 2:** The association between XPC-PAT, IVS11-6 and Lys939Gln among 1086 subjects

	**IVS 11-6**			
**XPC-PAT**	**C/C**	**C/A**	**A/A**	**Lys939Gln**
−/−	394	0	0	A/A
	0	3	0	A/C
	0	0	0	C/C
				
−/+	0	0	0	A/A
	1	517	0	A/C
	0	0	1	C/C
				
+/+	0	0	0	A/A
	0	1	0	A/C
	0	0	169	C/C

**Table 3:** The association between the XPC polymorphisms (XPC-PAT, Lys939Gln and IVS11-6) and bladder TCC risk

**Polymorphisms**	**Genotypes**	**Control^a^ (*n*)**	**Cases^a^ (*n*)**	**Crude OR (95% CI)**	**Adjusted OR (95% CI)**	***P*-value**
XPC-PAT	−/−	204	215	1.00	1.00	
	+/−	288	242	0.80 (0.62–1.03)	0.82 (0.63–1.07)	0.15
	+/+	85	87	0.97 (0.68–1.38)	0.98 (0.68–1.42)	0.95
						
Lys939Gln	A/A	192	204	1.00	1.00	
	A/C	285	241	0.80 (0.61–1.04)	0.82 (0.63–1.08)	0.14
	C/C	84	87	0.97 (0.67–1.42)	0.99 (0.69–1.43)	0.97
						
IVS11-6	C/C	196	206	1.00	1.00	
	A/C	287	243	0.81 (0.62–1.04)	0.83 (0.63–1.08)	0.16
	A/A	83	87	1.00 (0.70–1.43)	1.01 (0.70–1.46)	0.96

Adjusted OR: odds ratio adjusted for age, sex, smoking, occupational exposure and family history of bladder cancer; 95% CI=95% confidence interval.

a The total number of cases and controls vary between polymorphisms because of failure of genotyping.

**Table 4:** Stratified analysis of XPC polymorphisms (XPC-PAT) and bladder TCC risk by smoking status and occupational exposure

**XPC-PAT**	**Genotypes**	**Control (*n*)**	**Cases^a^ (*n*)**	**Crude OR (95% CI)**	**bOR (95% CI)**	***P*-value**
*Smoking status*						
Nonsmoker	−/−	64	46	1.00	1.00	
	+/−	105	55	0.73 (0.44–1.20)	0.67 (0.40–1.13)	0.14
	+/+	26	20	1.07 (0.53–2.15)	1.05 (0.51–2.17)	0.89
Ex and current smoker	−/−	140	168	1.00	1.00	
	+/−	183	187	0.85 (0.63–1.15)	0.87 (0.64–1.18)	0.38
	+/+	59	67	0.95 (0.63–1.43)	0.96 (0.63–1.46)	0.84
						
*Occupational exposure*						
No	−/−	169	151	1.00	1.00	
	+/−	243	180	0.83 (0.62–1.11)	0.82 (0.61–1.11)	0.21
	+/+	69	64	1.04 (0.69–1.56)	1.08 (0.71–1.63)	0.73
Yes	−/−	35	64	1.00	1.00	
	+/−	45	62	0.75 (0.43–1.32)	0.72 (0.40–1.27)	0.25
	+/+	16	23	0.79 (0.37–1.68)	0.76 (0.35–1.65)	0.49

a One case was excluded because of missing data on smoking status.

b OR=odds ratio adjusted for age, sex, occupational exposure and family history of bladder cancer (smoking status); OR=odds ratio adjusted for age, sex, smoking status and family history of bladder cancer (occupational exposure).

95% CI=95% confidence interval.
